# *α*-Lipoic Acid-Plus Ameliorates Endothelial Injury by Inhibiting the Apoptosis Pathway Mediated by Intralysosomal Cathepsins in an *In Vivo* and *In Vitro* Endothelial Injury Model

**DOI:** 10.1155/2022/8979904

**Published:** 2022-04-12

**Authors:** Yang Wang, Dejun Bao, Yongfei Dong, Xiangpin Wei, Jian Yu, Chaoshi Niu

**Affiliations:** ^1^Department of Neurosurgery, The First Affiliated Hospital of USTC, Division of Life Sciences and Medicine, University of Science and Technology of China, No. 17 Lujiang Road, Hefei, 230001 Anhui Province, China; ^2^Anhui Province Key Laboratory of Brain Function and Brain Disease, No. 17 Lujiang Road, Hefei, 230001 Anhui Province, China

## Abstract

*α*-Lipoic acid-plus (LAP), an amine derivative of *α*-lipoic acid, has been reported to protect cells from oxidative stress damage by reacting with lysosomal iron and is more powerful than desferrioxamine (DFO). However, the role of LAP in experimental carotid artery intimal injury (CAII) has not yet been well investigated. Therefore, we sought to uncover the role and potential endovascular protective mechanisms of LAP in endothelial injury. *In vitro*, oxyhemoglobin (OxyHb) stimulation of cultured human umbilical vein endothelial cells (HUVECs) simulated intimal injury. *In vivo*, balloon compression injury of the carotid artery was used to establish a rat CAII model. We found that the protein levels of cathepsin B/D, ferritin, transferrin receptor (TfR), cleaved caspase-3, and Bax increased in the injured endothelium and HUVECs but were rectified by DFO and LAP treatments, as revealed by western blotting and immunofluorescence staining. Additionally, DFO and LAP decreased oxidative stress levels and endothelial cell necrosis of the damaged endothelium. Moreover, DFO and LAP significantly ameliorated the increased oxidative stress, iron level, and lactic dehydrogenase activity of HUVECs and improved the reduced HUVEC viability induced by OxyHb. More importantly, DFO and LAP significantly reduced mitochondrial damage and were beneficial for maintaining lysosomal integrity, as indicated by acridine orange (AO), Lyso-Tracker Red, JC-1, and ATPB staining in HUVECs. Finally, LAP might offer more significant endovascular protective effects than DFO. Our data suggested that LAP exerted endovascular protective effects by inhibiting the apoptosis signaling pathway mediated by intralysosomal cathepsins by reacting with excessive iron in endothelial lysosomes after intimal injury.

## 1. Introduction

Carotid artery (CA) stenosis is a familiar and recognized risk factor for ischemic stroke, accounting for approximately 10–20% of strokes or transient ischemic attacks [1]. Although precautionary medical treatment has increased, the proportion of patients requiring surgical intervention remains high [2]. Both open operation and interventional surgery can induce different degrees of vascular injury. In brief, the repair process of blood vessels generally includes injured site reendothelialization, vascular remodeling, and intimal hyperplasia (IH) [3, 4]. Excessive IH is the primary cause of failure of vascular interventional therapy and hemadostenosis [5]. Endothelial cell (EC) injury has been identified as the first step toward postoperative IH [6] because the injured endothelial area is not capable of generating antiproliferative factors. The damaged area increases the risk of thrombus formation because of the coverage of platelets, fibrinogen, circulating red blood cells, and macrophages that release thrombotic factors and growth factors. Erythrocyte metabolites, especially the release of catalytically redox-active iron, have toxic effects, which are often neglected by investigators [7]. Reendothelialization relying on the proliferation and migration of ECs is a key process in vascular healing. Hence, the stimulation of EC proliferation and migration is essential to accelerate endothelial healing and restore vascular function in response to damaged ECs resulting from intimal injury [8]. Delayed reendothelialization induced by EC apoptosis and degeneration is one of the principal causes of excessive IH.

Iron is essential in the synthesis of hemoglobin and participates in mitochondrial respiration. Nevertheless, excessive iron intake can lead to damage to cells, organs, and the entire body [9]. Furthermore, iron increases reactive oxygen species (ROS) production and promotes calcium influx into mitochondria, which can disturb mitochondrial respiratory function and ultimately result in a decline in mitochondrial membrane potential (*ΔΨ*m) and mitochondrial damage. A previous study found that iron treatment significantly increased apoptotic cells and endothelial microparticles (EMPs) [10]. Iron overload is also involved in a number of cardiovascular and endothelial injury events [11]. Excessive free radical generation mediated by iron overload induces the loss of tight junction proteins and EC degeneration, leading to the opening of the blood–brain barrier (BBB) after transient forebrain ischemia [12]. Because iron induces EMP generation and EC apoptosis in association with increased oxidative stress, iron overload has been identified as a risk factor for cardiovascular events [13]. Hence, we designed this experiment to investigate the effects of redox-active iron on the endothelium *in vivo*, EC status *in vitro*, and the relevant damage mechanisms.

**Figure 1:**
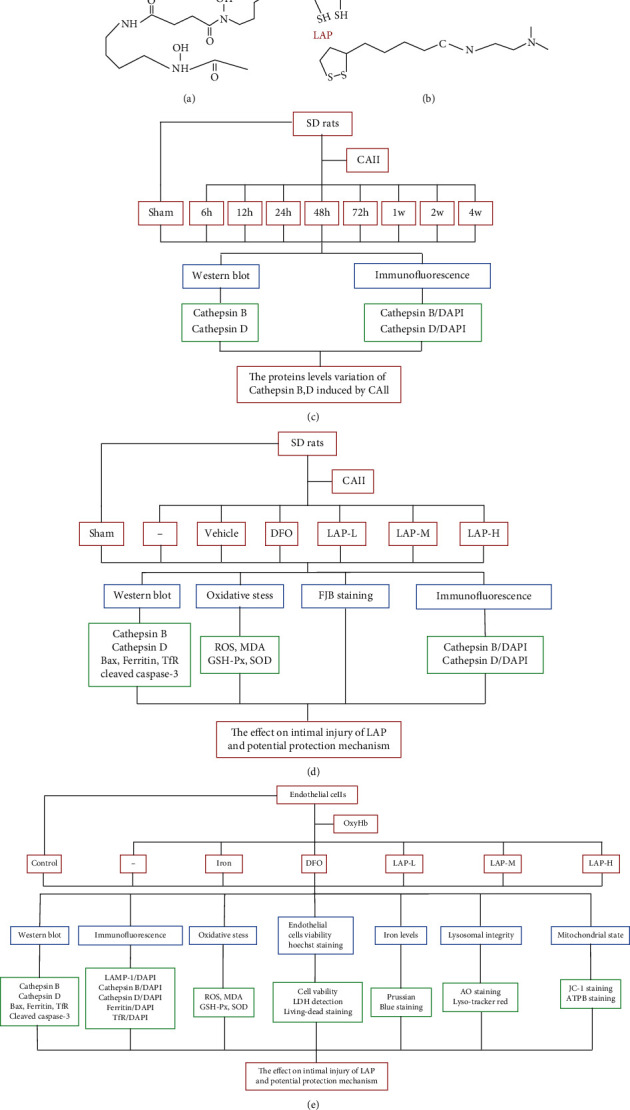
Structure of DFO, LAP, and DHLAP and experimental design. (a) Structure of DFO. (b) Structure of LAP and DHLAP. (c) Experiment 1 was designed to determine the involvement of cathepsin B/D under CAII conditions. (d) Experiment 2 was designed to explore the mechanisms of LAP in alleviating endothelial injury induced by CAII. (e) Experiment 3 was designed to explore the role and mechanisms of LAP in alleviating HUVEC injury induced by OxyHb *in vitro*.

As a radical scavenger and an iron-chelating agent, *α*-lipoic acid-plus (LAP) has already been confirmed to have neuroprotective effects in rat subarachnoid hemorrhage (SAH) models resulting from playing a stronger role in resisting oxidative stress by chelating iron than desferrioxamine (DFO). It has been demonstrated in the literature that LAP with a weak base (pKa = 8.0) can more easily enter lysosomes (pH = 4.6–5.0) through the differences in pH between the cytoplasm and lysozymes. Furthermore, the reduced forms of LAP (DHLAP) can provide sulfhydryl to react with redox-active iron [14, 15]. The structures of DFO, LAP, and DHLAP are shown in Figures 1(a) and 1(b). Although LAP has been shown to protect neuronal cells from oxidative stress injury triggered by labile iron *in vivo* and alleviate early brain injury after SAH, it has not been fully investigated whether LAP could inhibit endovascular injury induced by balloon compression, and the underlying mechanisms of action are unknown. Therefore, in this study, we explored the role of LAP in the reduction of EC apoptosis through the regulation of oxidative stress to identify novel medication for treating carotid artery intimal injury (CAII).

## 2. Methods

### 2.1. Experimental Animals

The experiment was approved by the Ethics Committee of the First Affiliated Hospital of the University of Science and Technology of China and was conducted in accordance with the National Institutes of Health regulations on animal feeding and application. Adult male Sprague–Dawley (SD) rats weighing approximately 300 g were supplied by Zhaoyan New Drug Research Center (Suzhou, China) Co., Ltd. The animals were housed in a constant humidity and temperature environment and were fed food and water regularly.

### 2.2. Experimental Designs and Drug Interventions

Experiment 1 was designed to demonstrate the role of cathepsin B/D in the endothelium following CAII. In experiment 1, 54 male adult rats were stochastically divided into nine point-in-time groups (*n* = 6): sham group; 6-hour, 12-hour, 24-hour, 48-hour, and 72-hour group; and 1-week, 2-week, and 4-week CAII groups. The CAs were collected from sham and CAII rats at various time points for western blotting and immunofluorescence (IF) assays. The experimental procedure is outlined in Figure 1(c). Experiment 2 was designed to explore the mechanisms of LAP in alleviating endothelial injury induced by CAII. In experiment 2, 84 male adult SD rats were stochastically divided into seven groups (*n* = 12): sham, CAII, CAII+vehicle, CAII+DFO (25 mg/kg), and CAII+LAP (100 mg/kg, 150 mg/kg, and 200 mg/kg). The animals were treated with LAP mixed with 0.5% methylcellulose through oral administration. DFO was administered through an intraperitoneal injection. DFO and LAP were administered 4 h after CAII induction, and both treatments were continued two more times. The doses of DFO and LAP were based on previous studies [15, 16]. Six rat CAs in every group were cut into slices and used for IF and Fluoro-Jade B (FJB) staining analysis. The remaining rats were sacrificed and perfused, and then CA samples were cut for western blotting and oxidative stress evaluation. The experimental procedure is displayed in Figure 1(d). Experiment 3 was designed to explore the roles and mechanisms of LAP in alleviating human umbilical vein endothelial cell (HUVEC) injury induced by oxyhemoglobin (OxyHb) *in vitro*. In experiment 3, logarithmically growing HUVECs were divided into the control, OxyHb, and OxyHb (Iron, DFO, LAP-L, LAP-M, and LAP-H) groups. HUVECs were exposed to OxyHb (10 *μ*M) and treated with DFO (1 mM) or three concentrations of LAP (0.2 *μ*M, 0.3 *μ*M, and 0.4 *μ*M) for 24 h before performing the subsequent assays [14]. Following the treatments, living HUVECs were first collected for cell viability assays, lactate dehydrogenase assay (LDH), acridine orange (AO) staining, live–dead cell staining, Lyso-Tracker Red, measurement of mitochondrial membrane potential (MMP), and oxidative stress evaluation. Second, HUVECs were fixed with paraformaldehyde for IF differential staining. Total protein from HUVECs was collected for western blotting assays. The specific experimental procedures are displayed in Figure 1(e).

### 2.3. Establishment of Rat CA Balloon Injury Models

Rat CAII models were established based on the approach described in our previous study [17]. In brief, the rats were primarily fixed in a specific head frame, and the CAs were thoroughly dissected. Then, a catheter with a balloon (Medtronic Inc., Minneapolis, MN, USA) was inserted from the gap of the external CA and slipped into the common CA. The balloon was inflated to approximately 2 atm, and the injury was induced by rubbing the inner surface of the common CA back and forth three times. Then, the catheter and balloon were withdrawn, and the injured external CA was sutured tightly under the operating microscope. The 2 cm common CA injured region was cut for analysis.

### 2.4. Cell Culture and Treatment

We performed the procedures for HUVEC extraction and culture in accordance with the previous literature [18]. To assess the impact of LAP on injured HUVECs *in vitro*, the HUVECs were exposed to OxyHb, DFO, and LAP at a gradient concentration for 24 h before performing the subsequent experiments.

### 2.5. Western Blotting

Western blotting was conducted as previously described [19]. HUVECs and CAs were added to lysis buffer and ground. The lysates were centrifuged twice at 12,000 g for 10 min at 4°C. Subsequently, the supernatant was extracted, and the protein concentration was measured using an enhanced bicinchoninic acid (BCA) protein assay kit. Protein samples were loaded, separated, electrophoresed, and transferred onto nitrocellulose filter membranes. The membranes were blocked with 5% bovine serum albumin at room temperature for 1 h and incubated with primary antibodies against cathepsin B (1 : 2000, ab214428, Abcam, Eugene, OR, USA), cathepsin D (1 : 1000, #2284s, Cell Signaling Technology, Danvers, MA, USA), cleaved caspase-3 (1 : 1000, ab2302, Abcam), Bax (1 : 1000, ab182733, Abcam), ferritin (1 : 1000, ab75973, Abcam), and transferrin receptor (TfR) (1 : 1000, ab269513, Abcam) overnight at 4°C. Next, the membranes were washed three times and incubated with an anti-mouse or anti-rabbit horseradish peroxidase conjugated-linked-secondary antibody (1 : 3000; 7076S and 7074s, respectively, Cell Signaling Technology) at room temperature for 2 h. Finally, the protein bands were detected using a luminescent image analyzer (Clinx ChemiScope5300, Clinx Science Instruments, Shanghai, China). The protein levels were analyzed using ImageJ software and were normalized to the relative density of the sham or the control group.

### 2.6. IF Microscopy

The injured CAs underwent a series of processes, including excision, fixation, and embedding in paraffin. Then, the CAs were cut into 4 *μ*m thick sections and analyzed through IF staining. Homoplastically, the treated HUVECs were fixed in 4% paraformaldehyde. Next, as previously described [20], the sections and HUVECs were incubated with primary antibodies against cathepsin B (1 : 100, ab214428, Abcam), cathepsin D (1 : 200, #2284 s, Cell Signaling Technology), ferritin (1 : 50, ab75973, Abcam), and TfR (1 : 50, ab269513, Abcam) at 4°C overnight. After washing three times on the following day, the samples were then incubated with Alexa Fluor 488-conjugated donkey anti-rabbit IgG (H+L) (1 : 300, A32790, Invitrogen, Carlsbad, CA, USA), Alexa Fluor 555-conjugated donkey anti-rabbit IgG (H+L) (1 : 300, A32794, Invitrogen), Alexa Fluor 488-conjugated goat anti-mouse IgG (1 : 300, A-11001, Invitrogen), and Alexa Fluor 555-conjugated goat anti-mouse (1 : 300, A-21424, Invitrogen) secondary antibodies at 37°C for 1 h. Next, 4,6-diamino-2-phenylindole (SouthernBiotech, Birmingham, AL, USA) was added to each section for coverslipping. Finally, the sections were observed under a fluorescence microscope (OLYMPUS BX50/BX-FLA/DP70; Olympus Co., Tokyo, Japan), and ImageJ software was used to quantify the fluorescence intensity.

### 2.7. Prussian Blue Reaction

The intracellular iron level of HUVECs was evaluated through the Prussian blue reaction of living HUVECs as described in the manufacturer's protocols (G1422, Solarbio, Beijing, China). Pictures were obtained under a light microscope (×100) as described previously [21].

### 2.8. Oxidative Stress Indicator Assay

The concentrations of ROS (E004-1-1, Nanjing Jiancheng Bioengineering Institute, Jiangsu, China) and thiobarbituric acid reactive substances (TBARS) (A003-1-2) and the activities of superoxide dismutase (SOD) (A001-3-2) and glutathione peroxidase (GSH-Px) (A005-1-2) of the impaired CAs and HUVECs were measured as described in the manufacturer's procedures for the detection kits [22].

### 2.9. FJB Staining

FJB is a hypersensitive and specific fluorescent dye used to indicate HUVEC degradation, and the staining process was based on a previous literature [23]. Briefly, the CA sections were immersed in 0.06% potassium permanganate (KMnO_4_) solution shielded from light at room temperature for 15 min after dewaxing. Next, the sections were added and reacted with FJB working solution (in 0.1% acetic acid solvent) (AG310, Sigma–Aldrich, St. Louis, MO, USA) for 1 h after being washed. Then, the sections were air-dried in a fume hood. Finally, the stained sections were sealed with neutral balsam mounting medium and observed under a fluorescence microscope, and images were taken in parallel to count FJB-positive cells.

### 2.10. Cell Viability Assays

First, the treated HUVECs were fixed in 50% trichloroacetic acid and stained with a sulforhodamine B (SRB) solution (230162, Sigma–Aldrich) as described previously [24]. Next, the absorbance of SRB was measured at a wavelength of 565 nm using a microplate reader. The assay was performed in triplicate and repeated at least three times independently.

### 2.11. LDH Assay

Based on the manufacturer's protocol for the LDH assay kit (C0016; Beyotime Institute of Biotechnology, Shanghai, China), LDH activity measurement was conducted to evaluate the degree of damage to cultured HUVECs [25].

### 2.12. Live–Dead Cell Staining In Vitro

HUVEC death was examined by live–dead cell staining 48 h after OxyHb and iron interventions. A calcein-AM/propidium iodide double-stain kit (Thermo Fisher Scientific, Shanghai, China) was used to detect cultured HUVEC apoptosis in accordance with the manufacturer's protocol [26]. Primarily, the culture medium was removed, and living HUVECs were washed three times. Next, the preconfigured working reagent mixed with calcein-AM and propidium iodide was added to the HUVECs and incubated for 30 min at room temperature. Finally, the cell death rate was analyzed and counted under a fluorescence microscope.

### 2.13. Hoechst Staining

HUVEC apoptosis was examined by Hoechst staining 48 h after OxyHb and iron interventions. First, we added an appropriate amount of Hoechst dye (C1018; Beyotime Institute of Biotechnology) to each well of the 12-well plate, which had to fully cover the sample. Next, the cell culture was incubated at 37°C for 20–30 min. Finally, we conducted fluorescence detection after discarding the staining solution and washing it with culture medium two or three times. When cell apoptosis occurred, the nuclei of apoptotic cells were observed with dense staining or fragmented dense staining under a fluorescence microscope.

### 2.14. AO Staining

HUVECs at the logarithmic growth stage were plated into 24-well plates and cultured for 24 h. Then, the original culture medium was removed from each well, and 1 mL of culture medium containing OxyHb, DFO, and LAP was added to each well at the corresponding concentrations. After culturing for 24 h, the original culture medium in each well was removed, and the cells were washed with phosphate-buffered saline (PBS). Next, AO dye solution (A7847, Sigma–Aldrich) was added and reacted at room temperature for 1 min. Ultimately, the cells were observed, photographed, and recorded under a fluorescence microscope after removing PBS and washing the cells [27].

### 2.15. Lyso-Tracker Red

For lysosomal staining, 1 *μ*L of Lyso-Tracker Red solution was added to 20 mL of cell culture medium and mixed to form Lyso-Tracker Red working solution. Then, the cell culture medium was removed, and Lyso-Tracker Red working solution preincubated at 37°C was added. Next, the cells were incubated for 30–120 min at 37°C. Finally, the Lyso-Tracker Red working solution was removed, and a new cell culture solution was added. The lysosomes in the cytoplasm were stained with bright and intense fluorescence under fluorescence microscopy.

### 2.16. MMP Measurement

The MMP was detected by a JC-1 kit (Beyotime) based on the instruction manual [26]. First, the treated HUVECs cultured in 12-well plates were washed with PBS; then, JC-1 working solution was added and reacted in a dark room for 20 min. Finally, the cultured HUVECs were observed under a fluorescence microscope. The JC-1 fluorescence intensity ratio (red/green) was calculated for the MMP in HUVECs.

### 2.17. Statistical Analysis

GraphPad Prism 7.0 software (San Diego, CA, USA) was applied for data processing and analysis. The data are shown as the mean ± standard error of the mean (SEM). One-way or two-way analysis of variance (ANOVA) was applied for multiple comparisons. Tukey's *post hoc* tests were used for comparisons between two pairs in multiple groups. *P* < 0.05 was considered statistically significant.

## 3. Results

### 3.1. Cathepsin B/D Is Activated following CAII

**Figure 2:**
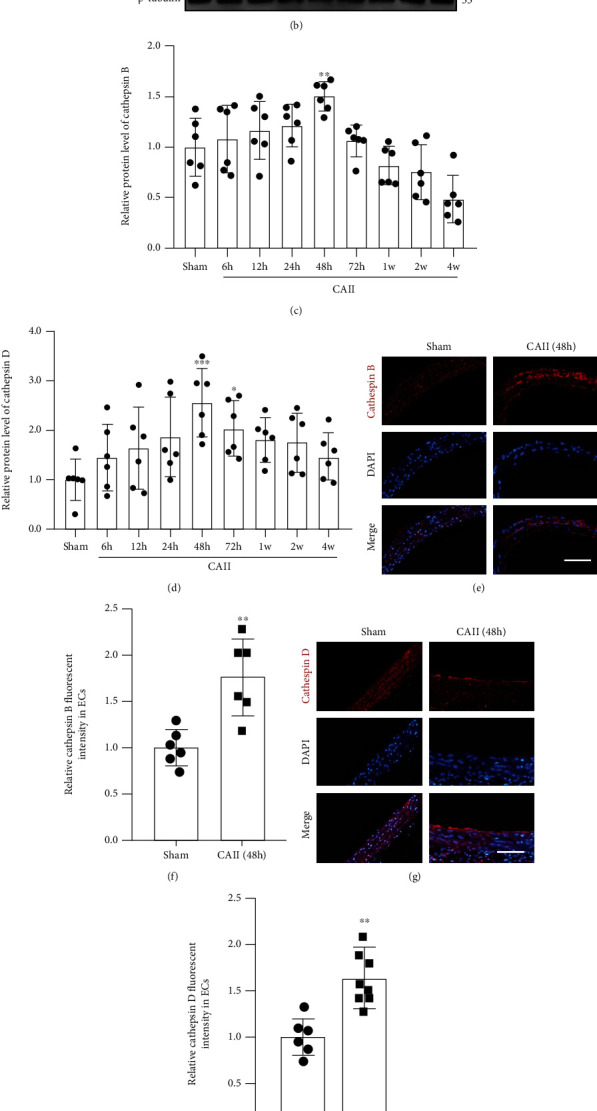
Time course of the protein levels of cathepsin B/D after CAII. (a, b) Western blot revealed the protein levels of cathepsin B/D at sham; 6, 12, 24, 48, and 72 h; and 1 week, 2 weeks, and 4 weeks after CAII. (c, d) Quantification of cathepsin B/D protein levels was normalized to the sham group. (e, g) IF analysis was performed with antibodies against cathepsin B/D (red) in CAII sections. Nuclei were labeled with DAPI (blue). Representative images of the sham group and CAII (48 h) group are shown. Scale bar, 100 *μ*m. (f, h) Immunopositivity of cathepsins B and D in the endothelium. The sham group was used as the standard. Data are shown as the mean ± SEM (*n* = 6). ^∗^*P* < 0.05,  ^∗∗^*P* < 0.01, and^∗∗∗^*P* < 0.001 relative to the sham group.

To detect changes in cathepsin B/D expression levels after CAII, we performed western blotting and IF staining. Western blotting results showed that cathepsin B/D expression levels increased after CAII, attained the highest peak at 48 h and then gradually recovered within 4 weeks (Figures 2(a)–2(d)) (^∗∗^*P* = 0.0055,  ^∗∗∗^*P* = 0.0007, and^∗^*P* = 0.0374). IF also showed that the immunopositivity of cathepsin B/D increased at each time point after SAH in comparison with the sham group (Figures 2(e)–2(h)) (^∗∗^*P* = 0.0021,  ^∗∗^*P* = 0.0020). The previous results indicated that cathepsins B and D may participate in the pathological process of CAII and are activated following CAII. Furthermore, 48 h was regarded as an optimal intervention point in further studies.

### 3.2. DFO and Different Concentrations of LAP Alleviate Cathepsin B/D, Cleaved Caspase-3, and Bax Expression Levels after Experimental CAII

**Figure 3:**
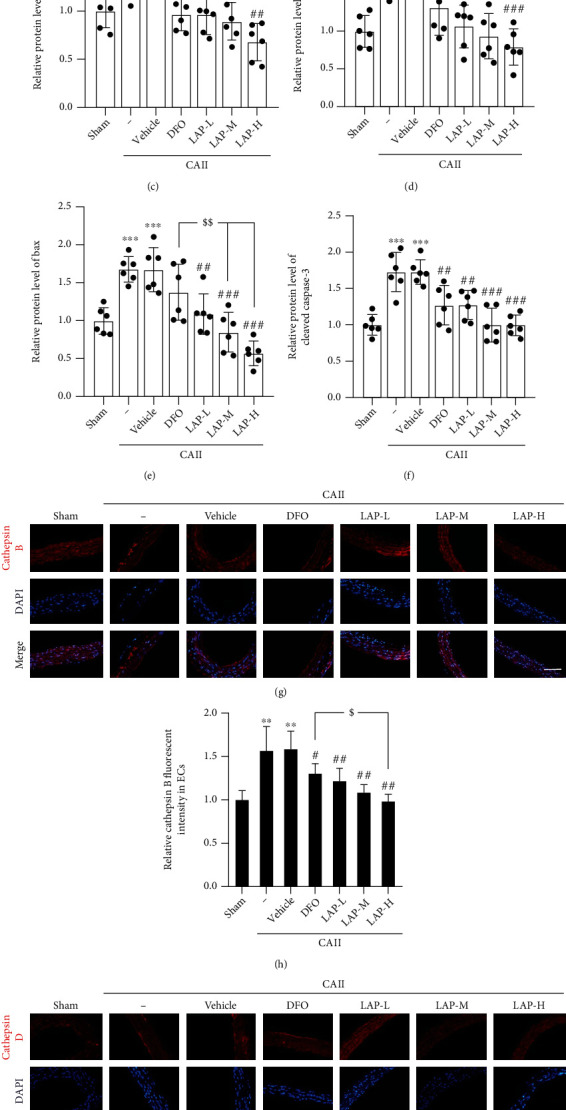
Changes in cathepsin B/D, cleaved caspase-3, and Bax after DFO and LAP treatments under CAII conditions. (a, b) Western blot revealed cathepsin B/D, cleaved caspase-3, and Bax expression levels in the endothelium. (c–f) The mean values of the protein levels of cathepsin B/D, cleaved caspase-3, and Bax in the sham group were normalized to 1.0. (g, i) IF analysis was performed using cathepsin B/D antibodies (red), and the nuclei were fluorescently labeled with DAPI (blue). (h, j) The mean value of the fluorescence intensity of the sham group was normalized to 1.0. Scale bar, 100 *μ*m. Data are shown as means ± SEM. ^∗^*P* < 0.05,  ^∗∗^*P* < 0.01, and^∗∗∗^*P* < 0.001*vs.* the sham group; ^#^*P* < 0.05,  ^##^*P* < 0.01, and^###^*P* < 0.001*vs.* CAII+vehicle group; ^$^*P* < 0.05 and ^$$^*P* < 0.01*vs.* CAII+DFO group; *n* = 6.

Western blotting was applied to assess target protein expression in the CA tissues after treatments with DFO and with different concentrations of LAP (Figures 3(a)–3(f)). The results showed that the expression levels of cathepsin B/D (^∗∗^*P* = 0.0037,  ^∗^*P* = 0.0103;  ^∗∗∗^*P* = 0.0006,  ^∗∗∗^*P* = 0.0004), cleaved caspase-3 (^∗∗∗^*P* = 0.00034,  ^∗∗∗^*P* = 0.00049), and Bax (^∗∗∗^*P* = 0.0002,  ^∗∗∗^*P* = 0.0003) in the CAII and CAII+vehicle groups were higher than those in the sham group. In contrast, the expression levels of cathepsin B/D (^##^*P* = 0.0038,  ^##^*P* = 0.0035,  ^##^*P* = 0.0030,  ^##^*P* = 0.0017;  ^##^*P* = 0.0012,  ^###^*P* = 0.0007,  ^###^*P* = 0.0005,  ^###^*P* = 0.0002), cleaved caspase-3 (^##^*P* = 0.0034, ^##^*P* = 0.0035,  ^###^*P* = 0.0006, and^###^*P* = 0.0005), and Bax (^##^*P* = 0.0015,  ^###^*P* = 0.0004, and^###^*P* = 0.0003) in the CAII+DFO and CAII+LAP groups were lower than those in the CAII+vehicle group. Note that LAP further attenuated cathepsin B/D (^$^*P* = 0.0362, ^$^*P* = 0.0197), cleaved caspase-3, and Bax (^$$^*P* = 0.0049, ^$$^*P* = 0.0024) expression levels compared with DFO (*P* < 0.05 or *P* < 0.01). Moreover, IF staining showed similar trends in the levels of cathepsin B/D, cleaved caspase-3, and Bax after CAII (Figures 3(g)–3(j)).

### 3.3. DFO and Different Concentrations of LAP Alleviate Cathepsin B/D, Cleaved Caspase-3, and Bax Expression Levels following HUVEC Injury

**Figure 4:**
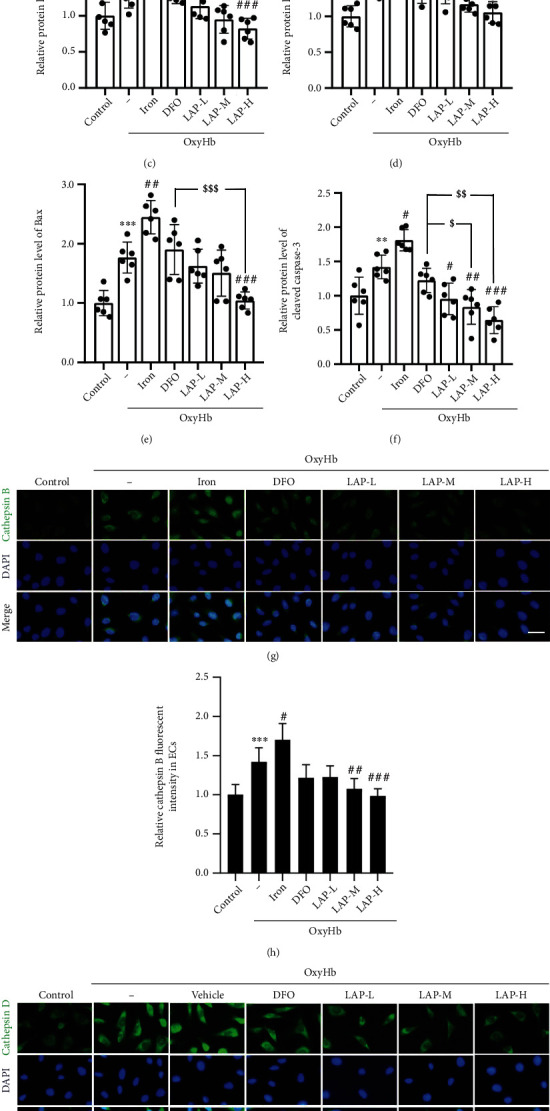
Changes in cathepsin B/D, cleaved caspase-3, and Bax after DFO and LAP treatments under HUVEC injury conditions. (a, b) Western blot showing cathepsin B/D, cleaved caspase-3, and Bax expression levels in HUVECs. (c–f) The mean values of the protein levels of cathepsin B/D, cleaved caspase-3, and Bax in the control group were normalized to 1.0. (g, i) IF analysis was performed using cathepsin B/D antibodies (green), and the nuclei were fluorescently labeled with DAPI (blue). (h, j) The mean value of the fluorescence intensity of the control group was normalized to 1.0. Scale bar, 100 *μ*m. Data are shown as the means ± SEM. ^∗^*P* < 0.05,  ^∗∗^*P* < 0.01, and^∗∗∗^*P* < 0.001*vs*. Control group; ^#^*P* <0.05, ^##^*P* <0.01, ^###^*P* <0.001 *vs*. OxyHb group; ^$^*P* < 0.05,  ^$$^*P* < 0.01, and^$$$^*P* < 0.001*vs*. OxyHb+DFO group; *n* = 6.

Western blotting was applied to assess target protein expression levels in HUVECs after treatment with iron, DFO, and different concentrations of LAP (Figures 4(a)–4(f)). The results showed that the expression levels of cathepsin B/D (^∗^*P* = 0.028 and^∗∗∗^*P* = 0.0002), cleaved caspase-3 (^∗∗^*P* = 0.0083), and Bax (^∗∗∗^*P* = 0.0005) in the OxyHb group were higher than those in the control group. In contrast, iron treatment further increased the expression levels of cathepsin B/D (^#^*P* = 0.0357 and ^###^*P* = 0.0005), cleaved caspase-3 (^#^*P* = 0.0154), and Bax (^##^*P* = 0.0019). Meanwhile, DFO and LAP decreased the expression levels of cathepsin B/D (^##^*P* = 0.0096, ^###^*P* = 0.0005; ^#^*P* = 0.0231, ^###^*P* = 0.0007), cleaved caspase-3 (^#^*P* = 0.0189, ^##^*P* = 0.0056, and ^###^*P* = 0.0007), and Bax (^###^*P* = 0.0009). Note that LAP further attenuated cathepsin B/D (^$$^*P* = 0.0095, ^$$$^*P* = 0.0005, and ^$^*P* = 0.0130), cleaved caspase-3 (^$^*P* = 0.0161, ^$$^*P* = 0.0086), and Bax (^$$$^*P* = 0.0003) expression levels relative to DFO. Moreover, immunofluorescent staining showed similar trends in cathepsin B/D, cleaved caspase-3, and Bax following HUVEC injury by OxyHb stimulation (Figures 4(g)–3(j)).

### 3.4. DFO and Different Concentrations of LAP Alleviate Oxidative Stress Levels under Endothelial and HUVEC Injury Conditions

**Figure 5:**
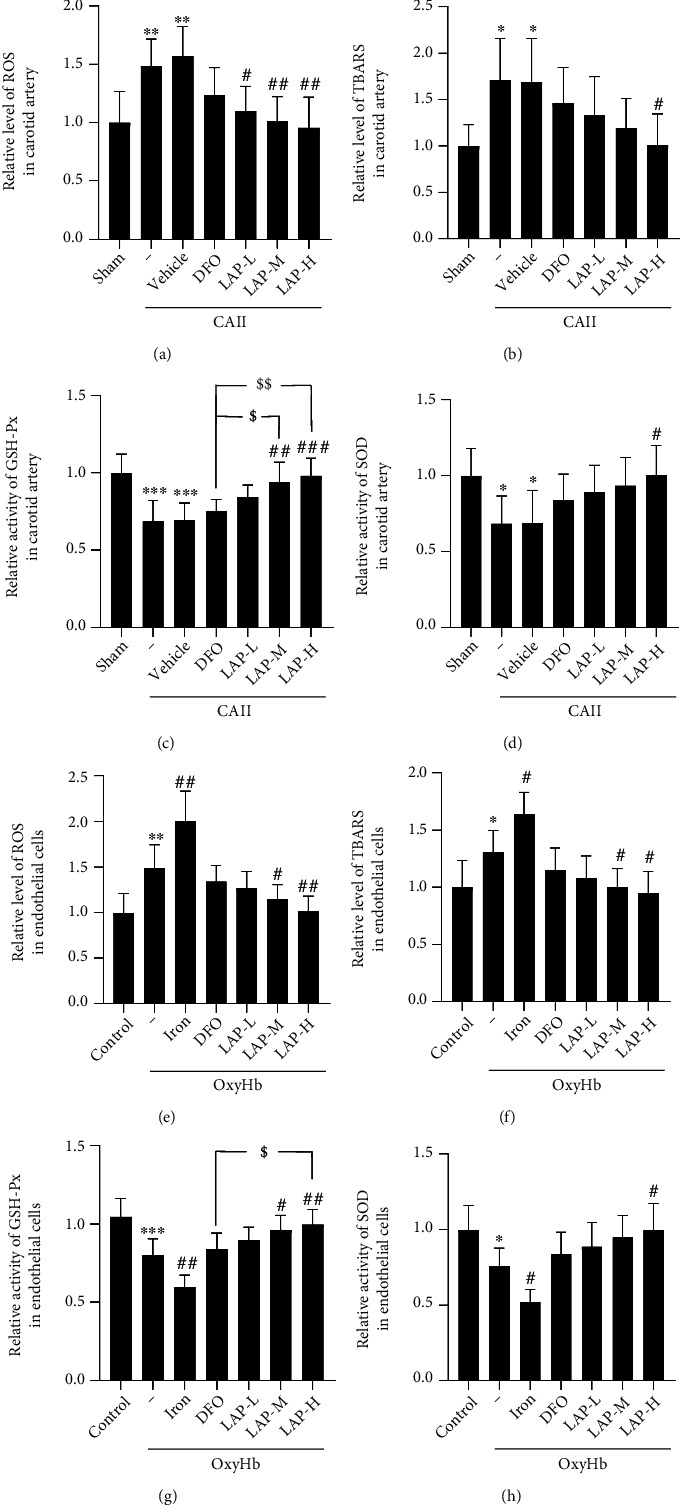
The variation in oxidative stress after DFO and LAP administration under endothelial and HUVEC injury conditions. Levels of oxidative stress after DFO and LAP administration in endothelial tissue (a–d) and HUVECs (e–h). The mean values of the levels of ROS and TBARS and the activities of SOD and GSH-Px in the sham and control groups were normalized to 1.0. Data are shown as the means ± SEM. ^∗^*P* < 0.05,  ^∗∗^*P* < 0.01, and^∗∗∗^*P* < 0.001*vs*. sham and control groups; ^#^*P* < 0.05,  ^##^*P* < 0.01, and^###^*P* < 0.01*vs*. CAII+vehicle and OxyHb groups; ^$^*P* < 0.05 and^$$^*P* < 0.01*vs*. CAII+DFO group and OxyHb+DFO group; *n* = 6.

The results of the assay for oxidative stress indexes in CA tissue and HUVECs are displayed in Figures 5(a)–5(d) and Figures 5(e)–5(h), respectively. The concentrations of ROS (^∗∗^*P* = 0.0061) and TBARS (^∗^*P* = 0.0170) in the CA samples in the CAII+vehicle group were markedly higher than those in the Sham group (*P* < 0.05 or *P* < 0.01), whereas DFO and LAP treatments both significantly restrained the increase in ROS (^#^*P* = 0.0373,  ^##^*P* = 0.0078, and^##^*P* = 0.0026) and TBARS (^#^*P* = 0.0194) concentrations in CAs. In contrast, CAII caused an obvious decline in SOD (∗∗∗*P* =0.0002) and GSH-Px (∗*P* = 0.0332) activities in CAs, whereas LAP inhibited the reduction of SOD (^##^*P* = 0.0024, ^###^*P* = 0.0004) and GSH-Px (^#^*P* = 0.0300) activities. The average levels of ROS (^∗∗^*P* = 0.0023) and TBARS (^∗^*P* = 0.0432) in the HUVECs in the OxyHb+vehicle group were markedly higher than those in the Control group. Moreover, iron treatment further increased the average concentrations of ROS (^##^*P* = 0.0011) and TBARS (^#^*P* = 0.0306) under OxyHb treatment conditions. In contrast, LAP treatment delayed the increase in ROS (^#^*P* = 0.0477, ^##^*P* = 0.0034) and TBARS (^#^*P* = 0.0424, ^#^*P* = 0.0155) concentrations in the HUVECs. Conversely, OxyHb caused a distinct depletion of SOD (^∗∗∗^*P* = 0.0005) and GSH-Px (^∗^*P* = 0.0311) activities in HUVECs. In contrast, iron treatment further decreased SOD (^##^*P* = 0.0032) and GSH-Px (^#^*P* = 0.0354) activities in HUVECs, whereas LAP was obviously able to restrain the decline in SOD (^#^*P* = 0.0301, ^##^*P* = 0.0064) and GSH-Px (^#^*P* = 0.0339) activities.

### 3.5. LAP Rescues Damaged HUVECs In Vitro

**Figure 6:**
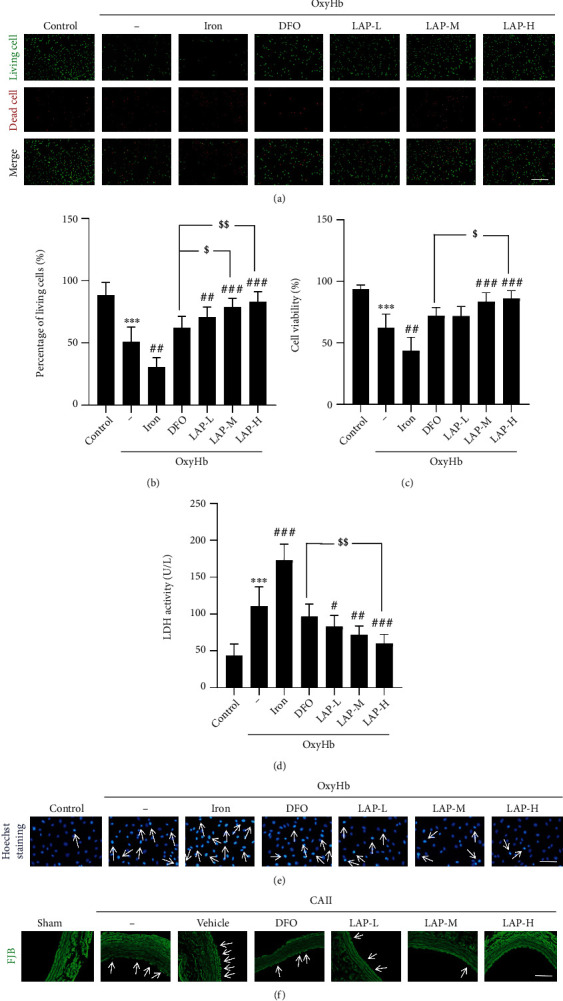
LAP attenuated cellular damage and improved cellular viability *in vitro*. (a) Live–dead cellular staining: green staining indicates viable cells, and red staining indicates dead cells. Scale bar, 250 *μ*m. (b) The percentage of living cells revealed by live–dead cellular staining. (c) Cell viability was tested by the SRB assay in HUVECs. (d) Cell damage was tested by the LDH assay in the cultured HUVECs. (e) HUVEC apoptosis was evaluated by Hoechst staining. (f) EC degeneration in the endothelium 48 h after CAII was detected by FJB staining (green). Arrows point to apoptosis-positive and FJB-positive cells. Scale bar, 100 *μ*m. Data are shown as the means ± SEM. ^∗∗∗^*P* < 0.001*vs*. control group; ^#^*P* < 0.05,  ^##^*P* < 0.01, and^###^*P* < 0.001*vs*. OxyHb group; ^$^*P* < 0.05 and^$$^*P* < 0.01*vs.* OxyHb+DFO group; *n* = 6.

Live–dead cellular staining was applied to assess the survival rate of HUVECs (Figures 6(a) and 6(b)). The staining for live (green) and dead (red) cells indicated that OxyHb intervention significantly decreased the survival rate of HUVECs (^∗∗∗^*P* = 0.0004). However, the combined iron and OxyHb treatment group had a lower survival rate than the OxyHb group (^##^*P* = 0.0019). In contrast, the OxyHb+LAP-treated group had a higher survival rate than the OxyHb group (^##^*P* = 0.0023, ^###^*P* = 0.0007, and ^###^*P* = 0.0005). Meanwhile, the medium and high concentrations of LAP showed a more significant protective effect than DFO (^$^*P* = 0.0133, ^$$^*P* = 0.0015). The SRB assay was used to measure the viability of cells (Figure 6(c)). The results showed that OxyHb significantly decreased the viability of HUVECs (^∗∗∗^*P* = 0.0003), and iron treatment further decreased the viability of HUVECs after OxyHb treatment (^##^*P* = 0.0018). The cell viability in the OxyHb+LAP-treated group was markedly higher than that in the OxyHb group (^###^*P* = 0.0003, ^###^*P* = 0.0002). It was apparent that a high concentration of LAP further increased the viability of HUVECs compared with DFO (^$^*P* = 0.0230). Consistently, the LDH assay was used to measure the damage to HUVECs (Figure 6(d)). OxyHb increased the activity of LDH in comparison with the control group (^∗∗∗^*P* = 0.0005), and iron treatment further decreased the activity of LDH after OxyHb treatment (^###^*P* = 0.0003). The activity of LDH in the OxyHb+LAP-treated group markedly decreased (^#^*P* = 0.0476, ^##^*P* = 0.0026, and ^###^*P* = 0.0001) compared with that in the OxyHb group. The high concentration of LAP further decreased the activity of LDH compared with DFO (^$$^*P* = 0.0052). Moreover, Hoechst staining was used to measure HUVEC apoptosis (Figure 6(e)). OxyHb increased HUVEC apoptosis in comparison with the control group, and iron treatment further increased HUVEC apoptosis under OxyHb treatment, whereas DFO and different concentrations of LAP obviously decreased HUVEC apoptosis. In addition, FJB staining showed almost no FJB-positive cells in the sham group (Figure 6(f)), whereas the number of FJB-positive cells in the CAII group was significantly higher than that in the sham group. Compared with the CAII+vehicle group, the number of FJB-positive cells was significantly attenuated by DFO and LAP. Together, these results suggested that LAP had more significant vascular protective effects against DFO.

### 3.6. LAP Promotes Mitochondrial Transport and Distribution in Damaged HUVECs

**Figure 7:**
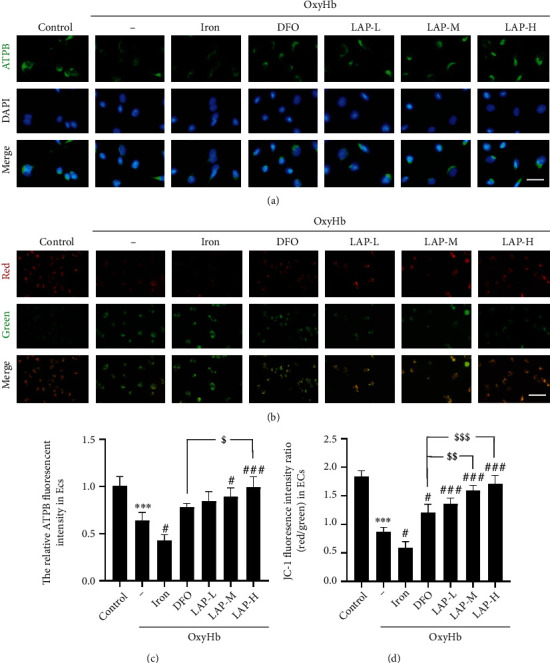
Effect of DFO and LAP treatments on mitochondrial damage under HUVEC injury conditions. (a) IF analysis was performed using ATPB antibody (green), and the nuclei were fluorescently labeled with DAPI (blue). (b) Alterations in mitochondrial membrane potential (*ΔΨ*m). The presence of red fluorescence represents normal *ΔΨ*m and a healthy state of the cells. Green fluorescence represents reduced *ΔΨ*m, and the cells are most likely in the early stage of apoptosis. (c) The mean value of the fluorescence intensity of the control group was normalized to 1.0. (d) JC-1 IF intensity ratio (red/green) in HUVECs. Scale bar, 100 *μ*m. Data are shown as the means ± SEM. ^∗∗∗^*P* < 0.001*vs*. control group; ^#^*P* < 0.05 and^###^*P* < 0.001*vs.* OxyHb group; ^$^*P* < 0.05,  ^$$^*P* < 0.01, and^$$$^*P* < 0.001*vs*. OxyHb+DFO group; *n* = 6.

The IF intensity of the mitochondrial marker ATPB was used to evaluate mitochondrial survival in HUVECs damaged by OxyHb stimulation (Figures 7(a) and 7(c)). The results showed that OxyHb significantly decreased the IF intensity of ATPB (^∗∗∗^*P* = 0.0005). Iron treatment further decreased the IF intensity of ATPB after OxyHb treatment (^#^*P* = 0.0468). Compared with that in the OxyHb group, the immunofluorescence intensity of ATPB in the OxyHb+LAP-treated group was markedly increased (^#^*P* = 0.0149, ^###^*P* = 0.0009). The high concentration of LAP further increased ATPB expression in HUVECs compared with DFO (^$^*P* = 0.0395). To study the effect of LAP on mitochondrial damage, we stained HUVECs *in vitro* with JC-1. In healthy mitochondria of HUVECs, JC-1 was shown by red fluorescence, whereas JC-1 transformed into green fluorescence in damaged mitochondria of HUVECs (Figures 7(b) and 7(d)). The results revealed that the percentage of green fluorescence-positive signals in the OxyHb group was brighter than that in the control group (^∗∗∗^*P* = 0.0002), whereas iron treatment further increased the percentage of green fluorescence-positive signals (^#^*P* = 0.0409). In contrast, in the OxyHb+DFO- and OxyHb+LAP-treated groups, the percentage of green-fluorescent-positive signal was lower than that in the OxyHb group (^#^*P* = 0.0134, ^###^*P* = 0.0005, ^###^*P* = 0.0003, and ^###^*P* = 0.0002). We also found that medium and high concentrations of LAP decreased the percentage of green-fluorescent-positive signals compared with DFO (^$$^*P* = 0.0046, ^$$$^*P* = 0.0004).

### 3.7. DFO and Different Concentrations of LAP Alleviate Ferritin and TfR Expression Levels and Iron Deposition following Endothelial and HUVEC Injury

**Figure 8:**
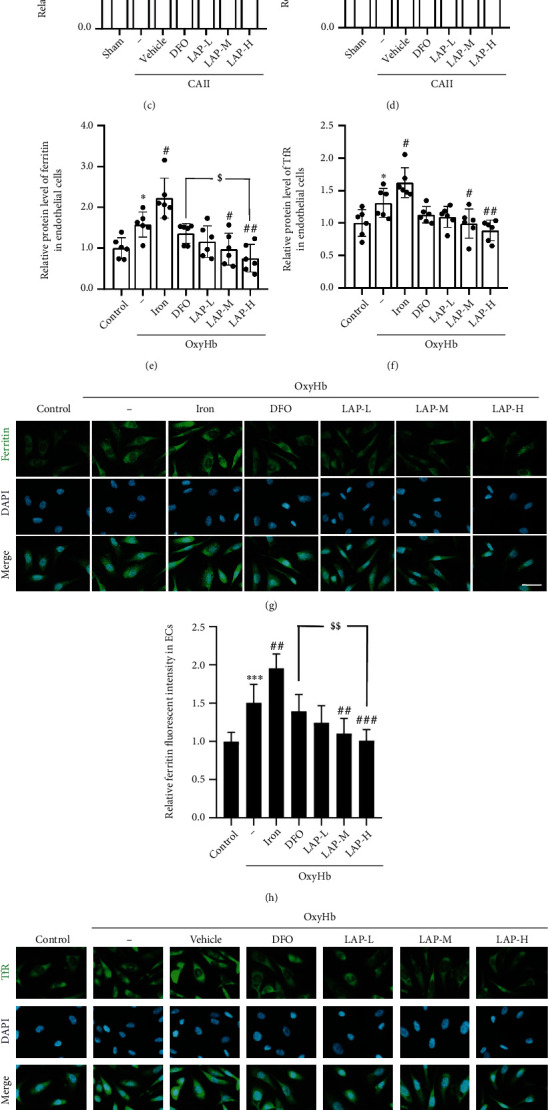
Changes in ferritin and TfR after DFO and LAP treatments under endothelial and HUVEC injury conditions. (a) Western blotting indicating ferritin and TfR expression levels in the endothelium. (b) Western blotting showing Ferritin and TfR expression levels in HUVECs. (c–f) The mean values of Ferritin and TfR protein levels in the sham and control groups were normalized to 1.0. (g, i) IF analysis was performed using Ferritin and TfR antibodies (green), and nuclei were fluorescently labeled with DAPI (blue). (h, j) The mean value of the fluorescence intensity of the control group was normalized to 1.0. (k) Iron deposition in the HUVECs was exhibited in different groups. Representative images are indicated. Scale bar, 100 *μ*m. Data are shown as the means ± SEM. ^∗^*P* < 0.05 and^∗∗∗^*P* < 0.001 vs. sham group and control group; ^#^*P* < 0.05,  ^##^*P* < 0.01, and^###^*P* < 0.001*vs*. CAII+vehicle group and OxyHb group; ^$^*P* < 0.05,  ^$$^*P* < 0.01, and^$$$^*P* < 0.001*vs*. CAII+DFO group and OxyHb+DFO group; *n* = 6.

Western blotting was applied to assess ferritin and TfR expression in HUVECs after treatment with DFO and different concentrations of LAP (Figures 8(a)–8(f)). The results showed that ferritin (^∗∗∗^*P* = 0.0006,  ^∗∗∗^*P* = 0.0004, and^∗^*P* = 0.0362) and TfR (^∗∗∗^*P* = 0.0002,  ^∗∗∗^*P* = 0.0002, and^∗^*P* = 0.0423) expression levels in the CAII and OxyHb groups increased relative to the sham and control groups, respectively. Iron treatment further increased the levels of ferritin (^#^P = 0.0153) and TfR (^#^*P* = 0.0392) in HUVECs. However, DFO and LAP decreased the levels of ferritin (^###^*P* = 0.0008, ^###^*P* = 0.0005; ^#^*P* = 0.0272, ^##^*P* = 0.0015) and TfR (^#^*P* = 0.0162, ^##^*P* = 0.0023, ^###^*P* = 0.0003; ^#^*P* = 0.0346, ^##^*P* = 0.0030) in the endothelium and HUVECs, respectively. Note that the medium and high concentrations of LAP further attenuated ferritin (^$$$^*P* = 0.0007, ^$$$^*P* = 0.0005, and ^$^*P* = 0.0249) and TfR (^$$^*P* = 0.0022) expression levels compared with DFO. Moreover, IF staining showed similar trends in ferritin and TfR expression levels following HUVEC injury (Figures 8(g)–8(j)). We performed Prussian blue reactions to assess the iron content in HUVECs (Figure 8(k)). In the control group, little iron deposition was observed in the HUVECs, whereas in the OxyHb group, significant iron deposition was found. Compared with the OxyHb group, iron deposition in HUVECs was markedly increased by iron addition and reduced by DFO and oral administration of LAP. In addition, the high concentration of LAP presumably exerted an additional effect on restraining iron deposition compared with DFO.

### 3.8. LAP Protects Lysosomes from Rupture

**Figure 9:**
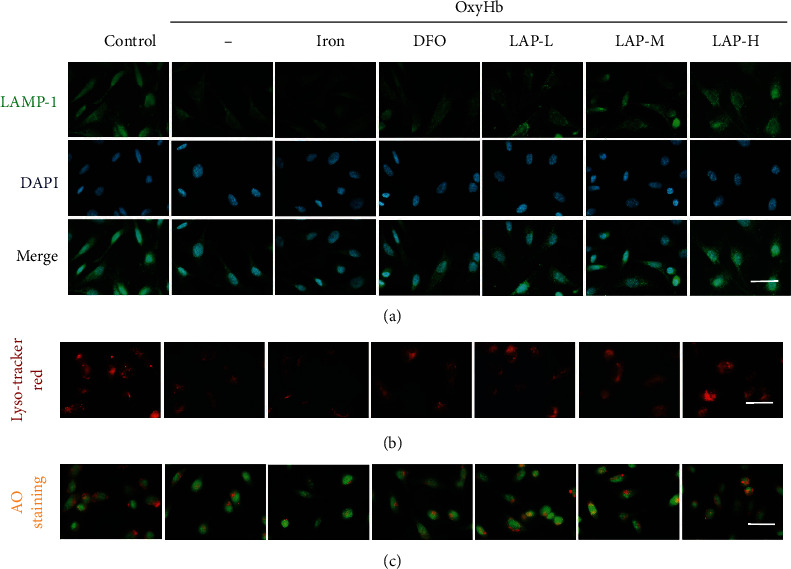
Effect of DFO and LAP treatments on lysosomal integrity following HUVEC injury. (a) IF analysis was performed using LAMP-1 antibody (green), and the nuclei were fluorescently labeled with DAPI (blue). (b) Lyso-Tracker Red cellular staining: red staining indicates intact lysosomes in HUVECs. (c) AO staining: orange staining indicates acidic compartment accumulation in HUVECs. Scale bar, 100 *μ*m.

To evaluate the quantity and state of lysosomes in HUVECs, we performed IF staining with lysosomal associated membrane protein 1 (LAMP-1) attached to different groups (Figure 9(a)). We observed a distinct reduction in LAMP-1 expression in the OxyHb group, and iron treatment further reduced LAMP-1 expression. In contrast, DFO and different concentrations of LAP remarkably inhibited lysosome rupture based on the alteration of LAMP-1 fluorescence intensity in HUVECs. However, the high concentration of LAP possibly exerted a more positive impact on stabilizing the lysosomal membrane than low concentrations of LAP and DFO. As a preliminary indicator of the lysosomal state, the acidic compartments in HUVECs were observed by Lyso-Tracker Red staining and AO staining. As shown in Figures 9(b) and 9(c), the accumulation of acidic compartments in HUVECs in the control group was normal. The accumulation of acidic compartments in HUVECs significantly decreased compared with that in the control group following OxyHb stimulation and iron treatment. The accumulation of acidic compartments in HUVECs, however, significantly increased after DFO and LAP treatments.

## 4. Discussion

**Figure 10:**
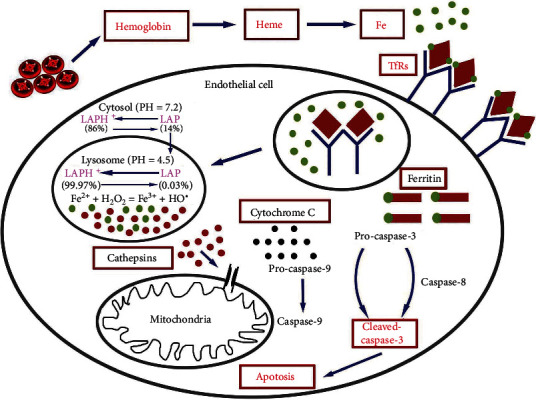
Potential mechanism by which LAP alleviates HUVEC and endothelial damage by inhibiting the mitochondrial apoptosis pathway triggered by lysosomal cathepsins.

In this study, we found that LAP exerts an endovascular protective effect by reacting with excess iron in endothelial lysosomes to inhibit the intimal injury-mediated apoptotic signaling pathway induced by intralysosomal cathepsins (Figure 10). As a lysosomotropic and iron-chelating agent, LAP can target and gather in lysosomes and inhibit the Fenton reaction by reacting with active iron. Hence, LAP inhibited the apoptosis pathway mediated by mitochondria by reducing the generation of hydroxyl radicals, stabilizing the lysosome membrane, and decreasing the release of cathepsins. Ultimately, LAP reduced EC apoptosis and injury, promoted reendothelialization of the damaged area, inhibited excessive proliferation of the vascular intima, and reduced the rate of vascular restenosis.

Vascular endothelium damage caused by iron overload in association with oxidative stress and the underlying mechanisms have not yet been clarified. Prolonged exposure to iron enhances endothelial nicotinamide adenine dinucleotide phosphate (NADPH) oxidase activity by enhancing cellular iron, heme, p22phox protein levels, and p22phox gene transcription. DFO, a routine iron-chelating agent, may effectively reduce vascular inflammation and oxidative stress in atherosclerosis by suppressing endothelial NADPH oxidase activity [28]. In our experiment, DFO was also able to reduce oxidative stress in damaged vascular endothelium and HUVECs, but the most satisfactory result was not achieved; however, the effect of LAP resisting oxidative stress was more significant than DFO. In addition, iron could induce endothelial microparticle generation and apoptosis of ECs related to increased oxidative stress, and carvedilol could provide a protective effect based on these mechanisms [13]. Recent research findings have revealed that nobiletin can protect HUVECs from iron overload damage. The underlying mechanism could involve restraining the ROS/asymmetric dimethylarginine (ADMA)/dimethylarginine dimethylaminohydrolase 2 (DDAHII)/endothelial nitric oxide synthase (eNOS)/nitric oxide (NO) signaling pathway. Specifically, nobiletin can reduce oxidative stress, increase the expression and activity of DDAHII and NO production, decrease ADMA content, promote eNOS phosphorylation to maintain mitochondrial function, and protect the vascular endothelium against damage induced by iron overload [29]. In this study, the role of LAP is further clarified for LAP reacting directly with oxidized active iron compared to nobiletin. In transient forebrain ischemia rat models, iron overload and iron-mediated free radical production aggravated BBB injury by decreasing tight junction protein expression and increasing the degeneration of ECs [12]. Furthermore, iron overload induced ROS production and cultured vascular cell apoptosis, which aggravated atherosclerosis progression. Simultaneously, iron intake restriction or iron chelation therapy could suppress iron-aggravated atherosclerosis in ApoE-/- FPNwt/C326S mice [30]. Hence, iron overload aggravated vascular injury and affected vascular remodeling, and some drugs focusing on reducing iron overload will be investigated in depth soon. In our experiment, we found that iron treatment was able to increase oxidative stress levels and increase lysosomal membrane permeability. Thus, the leaked cathepsins from ruptured lysosomes activate the mitochondrial apoptotic pathway. Additional significant findings were that LAP reduced the content of ROS and TBARS and increased the activities of SOD and GSH-Px in the endothelium and HUVECs. These results showed that, in comparison with DFO, LAP possessed a more powerful ability to inhibit oxidative stress in lysosomes and cytoplasm by interacting with lysosomal iron.

Endothelial dysfunction is closely associated with IH, and several drugs have been discovered to inhibit endothelial dysfunction. A previous study found that anagliptin, a dipeptidyl peptidase IV (DPP-4) inhibitor, suppresses IH by preventing endothelial dysfunction and regulating SOD-1/RhoA/Jun N-terminal kinase- (JNK-) mediated EC migration following balloon-induced injury [31]. Moreover, finerenone, a novel mineralocorticoid receptor antagonist, apparently reduces EC apoptosis and smooth muscle cell proliferation, bringing about accelerated endothelial healing and delayed neointima formation of the injured vessels [32]. Furthermore, low-dose simvastatin can induce cardiac microvascular EC proliferation, migration, and antiapoptosis through the PI3K/Akt/mTOR/p70S6K and mTOR/FoxO3 signaling pathways. It then exerts a beneficial effect by regulating NO and ROS production in microvascular ECs [33]. Our research also confirmed that iron aggravated EC injury by aggravating lysosomal injury and that LAP could alleviate EC injury triggered by redox-active iron decomposed by OxyHb. Therefore, LAP possesses a more potent capacity to react with iron than DFO because of its reduced form and high concentration. In summary, we conclude that LAP is a more powerful antioxidant and is effective in stabilizing the lysosomal membrane by reacting with redox-active iron, which targets lysosomes.

Our present study has some limitations. First, we were not able to determine the precise concentration of intralysosomal iron in ECs relying on our current experimental platform. Second, we could not detect the oxidized or reduced LAP distribution within lysosomes in ECs. In summary, this research confirmed that LAP exerted a more potent endovascular protective effect than DFO. The underlying mechanism was that LAP restrained signaling pathways mediated by cathepsins through chelating intralysosomal and active iron. Consequently, drugs such as LAP, which target lysosomal iron, may have great therapeutic potential for treating endovascular injury.

## Data Availability

The data used to support the finding of this study are available from the corresponding author upon request.

## Abbreviations

ADMA: Asymmetric dimethylarginine
ANOVA: Analysis of variance
AO: Acridine orange
BBB: Blood–brain barrier
BCA: Bicinchoninic acid
CA: Carotid artery
CAII: Carotid artery intimal injury
DDAHII: Dimethylarginine dimethylaminohydrolase 2
DFO: Desferrioxamine
DHLAP: Reduced forms of LAP
DPP-4: Dipeptidyl peptidase IV
EC: Endothelial cell
EMP: Endothelial microparticle
eNOS: Endothelial nitric oxide synthase
FJB: Fluoro-Jade B
GSH-Px: Glutathione peroxidase
HUVEC: Human umbilical vein endothelial cell
IF: Immunofluorescence
IH: Intimal hyperplasia
JNK: Jun N-terminal kinase
LAMP: Lysosomal associated membrane protein 1
LAP: Lipoic acid-plus
LDH: Lactate dehydrogenase assay
TBARS: Thiobarbituric acid reactive substances
MMP: Mitochondrial membrane potential
mPTP: Mitochondrial permeability transition pore
NADPH: Nicotinamide adenine dinucleotide phosphate
NO: Nitric oxide
Oxy Hb: Oxyhemoglobin
PBS: Phosphate-buffered saline
ROS: Reactive oxygen species
SAH: Subarachnoid hemorrhage
SD: Sprague–Dawley
SEM: Standard error of the mean
SOD: Superoxide dismutase
SRB: Sulforhodamine B
TfR: Transferrin receptor.
