# Correction to “α‐Lipoic Acid‐Plus Ameliorates Endothelial Injury by Inhibiting the Apoptosis Pathway Mediated by Intralysosomal Cathepsins in an *In Vivo* and *In Vitro* Endothelial Injury Model”

**DOI:** 10.1155/omcl/9860825

**Published:** 2026-10-31

Y. Wang, D. Bao, Y. Dong, X. Wei, J. Yu, and C. Niu, “α‐Lipoic Acid‐Plus Ameliorates Endothelial Injury by Inhibiting the Apoptosis Pathway Mediated by Intralysosomal Cathepsins in an *In Vivo* and *In Vitro* Endothelial Injury Model,” *Oxidative Medicine and Cellular Longevity*, (2022): 8979904, https://doi.org/10.1155/2022/8979904.

In the article, the authors have identified errors in Figures 2 and 4, introduced during figure preparation. Specifically,

**Figure 2:**
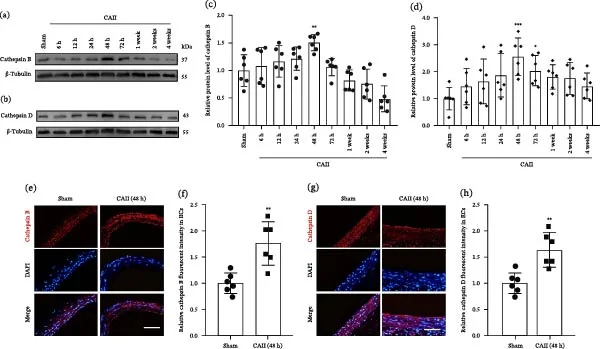
Time course of the protein levels of cathepsin B/D after CAII. (a, b) Western blot revealed the protein levels of cathepsin B/D at sham; 6, 12, 24, 48, and 72 h; and 1 week, 2 weeks, and 4 weeks after CAII. (c, d) Quantification of cathepsin B/D protein levels was normalized to the sham group. (e, g) IF analysis was performed with antibodies against cathepsin B/D (red) in CAII sections. Nuclei were labeled with DAPI (blue). Representative images of the sham group and CAII (48 h) group are shown. Scale bar, 100 μm. (f, h) Immunopositivity of cathepsins B and D in the endothelium. The sham group was used as the standard. Data are shown as the mean ± SEM (*n* = 6).  ^∗^ *p* < 0.05,  ^∗∗^ *p* < 0.01, and  ^∗∗∗^ *p* < 0.001 relative to the sham group.

**Figure 4:**
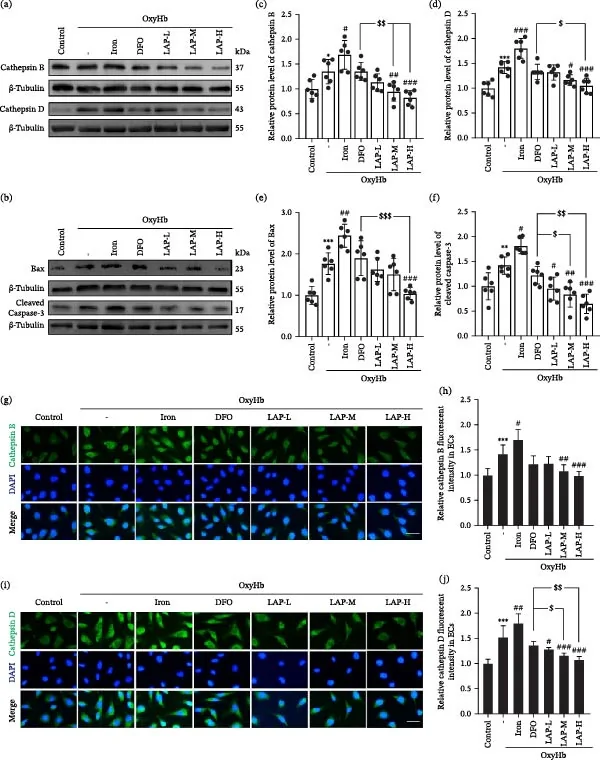
Changes in cathepsin B/D, cleaved caspase‐3, and Bax after DFO and LAP treatments under HUVEC injury conditions. (a, b) Western blot showing cathepsin B/D, cleaved caspase‐3, and Bax expression levels in HUVECs. (c–f) The mean values of the protein levels of cathepsin B/D, cleaved caspase‐3, and Bax in the control group were normalized to 1.0. (g, i) IF analysis was performed using cathepsin B/D antibodies (green), and the nuclei were fluorescently labeled with DAPI (blue). (h, j) The mean value of the fluorescence intensity of the control group was normalized to 1.0. Scale bar, 100 μm. Data are shown as the means ± SEM.  ^∗^ *p* < 0.05,  ^∗∗^ *p* < 0.01, and  ^∗∗∗^ *p* < 0.001 vs. Control group; ^#^ *p* < 0.05, ^##^ *p* < 0.01, ^###^ *p* < 0.001 vs. OxyHb group; ^$^ *p* < 0.05, ^$$^ *p* < 0.01, and ^$$$^ *p* < 0.001 vs. OxyHb+ DFO group; *n* = 6.

• In Figure 2e, the sham images appear the same as the LAP‐M images in Figure 3.
• In Figure 4b, the bottom β‐tubulin bands are the same as the β‐tubulin bands shown in Figure 8b.

The correct Figures 2 and 4 are shown below:

We apologize for these errors.

